# Evolving Lessons on Metazoan Primordial Germ Cells in Diversity and Development

**DOI:** 10.1002/mrd.70027

**Published:** 2025-05-11

**Authors:** Indrashis Bhattacharya, Lakshmi K. Nalinan, K. V. Anusree, Ahmed Saleel, Aditi Khamamkar, Souvik Dey

**Affiliations:** ^1^ Department of Zoology The Central University of Kerala Tejaswini Hills, Periye (PO) Kasaragod (DT) Kerala India; ^2^ Manipal Centre for Biotherapeutics Research Manipal Academy of Higher Education Manipal Karnataka India

**Keywords:** embryos, gonads, metazoan, primordial germ cell, sex determination, specification

## Abstract

Germ cells are pivotal for the continuation of biological species. The metazoan germline develops from primordial germ cells (PGCs) that undergo multiple rounds of mitotic divisions. The PGCs are specified by either maternal inheritance of asymmetrically polarized cytoplasmic mRNAs/proteins (found in roundworms, flies, fishes, frogs, and fowl) or via direct induction of epiblast cells from adjacent extraembryonic ectoderm in mammals. In all vertebrates, PGCs remain uncommitted to meiosis and migrate to colonize the developing gonadal ridge before sex determination. Multiple RNA‐binding proteins (e.g., *Vasa*, *Dnd*, *Dazl*, etc.) play crucial roles in PGC identity, expansion, survival, and migration. Postsex determination in mouse embryos, *Gata4*, expressing nascent gonads, induces *Dazl* expression in newly arriving germ cells that supports retinoic acid–mediated induction of meiotic onset. This article briefly discusses the developmental events regulating the PGC specification and commitment in metazoans. We also highlight the recent progress towards the in vitro generation of functional PGC‐like cells in rodents and humans.

## 1 Introduction

Primordial germ cells (PGCs) are the precursor/progenitor of the metazoan germline. The PGCs originate from developing embryonic tissues and expand mitotically (Extavour and Akam 2003). In roundworms, marine arrow worms (Chaetognatha), flies, teleost fishes, frogs, aspic viper snakes, and birds the PGCs develop continuously and undergo “*specification*” by germplasm (maternal ooplasm having asymmetrically localized messenger RNAs [mRNAs] and/or proteins) based segregation known as *preformation* (Strome and Updike 2015). However, in water bears or moss piglets (Tardigrades), grylloidean insects, salamander axolotls, and most reptiles and mammals, such an event remains discontinuous and PGC specification is achieved via an extrinsic induction process involving other cell types during larval/embryonic development, termed as *epigenesis* (Strome and Updike 2015). In most metazoans (except the nematode *Caenorhabditis elegans*), the specified PGCs exhibit direct migration towards the developing gonadal site (Richardson and Lehmann 2010). However, the migrating PGCs are found to be proliferative and uncommitted (retaining a broad developmental competence) towards meiotic fate in most of the vertebrates (Strome and Updike 2015).

**Figure 1:**
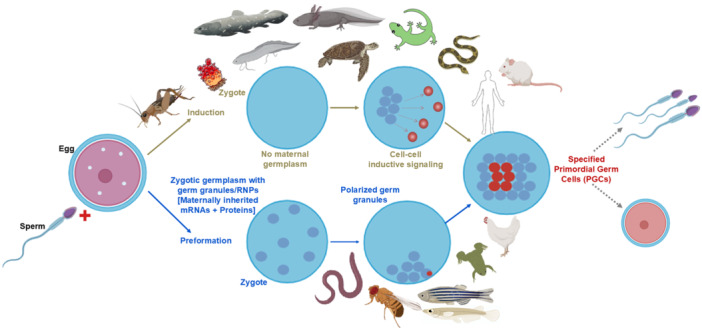
The mechanistic summary of two modes of PGC specification in metazoans. Preformative mode is based on asymmetrical localization of maternally inherited cytoplasmic mRNAs/proteins, found in roundworms, flies, fishes, frogs, and fowl, whereas inductive mode (through BMP signaling of adjacent cells) is exhibited by grylloidean insects, tunicates, coelacanth and lungfishes, salamanders, axolotls, most reptiles, and mammals. BMP, bone morphogenetic protein; mRNA, messenger RNA; PGC, primordial germ cell.

Figure 1 summarizes the mechanistic illustration of metazoan PGC specification. For example, acquiring somatic features by ectopically localized PGCs in fishes, namely, Dead end (*Dnd1*) depleted zebrafish PGCs get redirected to somatic fate (Strome and Updike 2015), or ectopically transplanted *Xenopus* PGCs (Lai et al. 2012) spontaneously differentiate into somatic cells. Finally, mammalian PGCs generate teratomas after ectopic transplantation (Stevens 1964) and show pluripotency in vitro (Matsui et al. 1992; Shamblott et al. 1998). In this article, we discuss the current concepts of the origin, specification, migration, and commitment of metazoan PGCs, emphasizing model organisms (viz., roundworms [*C. elegans*], fruit flies [*Drosophila melanogaster*], fishes [*Danio rerio*], frogs [*Xenopus laevis*], and mice [*Mus musculus*]) and humans. We also highlight the recent progress of generating PGC‐like cells (PGCLCs) in vitro with promising outcomes to achieve ex vivo gametogenesis.

## 2 Origin and Specification

PGCs are specified by maternal inheritance of nonuniformly localized ooplasmic mRNAs and RNA‐binding proteins (RBPs) found in roundworms, flies, fishes, frogs, and fowl. However, in mammals, such specification involves a direct induction of epiblast cells from adjacent extraembryonic ectoderm (EEE) (Extavour and Akam 2003; Strome and Updike 2015). Notably, most germplasm components are known as germ granules, which are amorphous, electron‐dense, cytosolic aggregates of RBPs (e.g., VASA‐related RNA helicases, Tudor‐domain proteins, NANOS, Argmethyltransferase PRMT5, Argonaute proteins, etc.) (Strome and Updike 2015).

*Roundworms*—In nematode worms (*C. elegans*), maternally inherited germline determinants localized to a single blastomere termed as germ or *“P granules”* (consisting of multiple mRNAs and RBPs) drive the specification of PGCs (Pazdernik and Schedl 2013). Intriguingly, although embryonic mislocalization of P granules does not lead to sterility during adulthood (Gallo et al. 2010), combined ablation of four RBPs (coded by Guanyl‐specific ribonuclease *Pgl1, Pgl3*, and adenosine triphosphate (ATP)‐dependent RNA helicase *Glh‐1, Glh‐4*) critical for P granule assembly, triggers somatic fate (Updike et al. 2014). Among the maternal mRNAs, maternal‐effect sterile (*Mes*) transcripts are essential for the development of the *C. elegans* germline (Capowski et al. 1991). Although homozygous *maternal‐effect sterile 1* (*Mes‐1*) mutants are fertile hermaphrodites, they reproduce sterile hermaphrodite offspring (Capowski et al. 1991). Mutants of *Mes‐1* show missegregated P granules that fail to form germ blastomeres (Strome et al. 1995). Similarly, the other four *Mes* genes, *Mes‐2* (homologs of the Polycomb group protein Enhancer of zeste), *Mes‐3* (novel protein without any recognizable motifs), *Mes‐4* (resembles *Mes‐2* with SET domain), and *Mes‐6* (homologs of the Polycomb protein extra sex combs) show sterile phenotypes due to germ‐cell apoptosis during larval development (Holdeman et al. 1998). However, worms with mutated *protein phosphatase 2A regulatory subunit 1* (*Pptr‐1*) (Gallo et al. 2010) and *maternal‐effect germ*‐*cell defective* (*Meg*) (J. T. Wang et al. 2014) exhibit unsegregated P granules without any fertility complications during adulthood. These data indicate that only the segregation of P granules to PGCs does not guarantee the germline specification in these taxa (Cassani and Seydoux 2024; Pazdernik and Schedl 2013). Notably, in adult worms, since mitotic divisions are induced by *Lag‐2* expressing distal tip cells, *Glp‐1/Lin‐12* expressing mitotic cells are restricted to the distal end, whereas meiotic cells appear more proximally (Francis et al. 1995; Kimble and Crittenden 2005). *Gld‐1* (defective in germline development 1) plays a critical role in oocyte development, as *Gld‐1* null (−/−) worms develop as hermaphrodites (Francis et al. 1995). In contrast, although *Gld‐2*, which is essential for meiotic prophase in both sexes, remains functionally redundant with *Gld‐1* for meiotic entry (Francis et al. 1995). Loss of either *Gld‐1* or *Gld‐2* in germ cells shows the normal meiotic entry; however, double mutant (*Gld‐1* [−/−] X *Gld‐2* [−/−]) germ cells fail to enter meiosis and form a tumor (Kadyk and Kimble 1998). Notably, *Glp‐1* signaling is considered to support mitosis by attenuation of *Gld‐1 and Gld‐2* functions (Francis et al. 1995).

*Insects*—Germ granules are necessary and sufficient to drive the germline specification in *Drosophila* (Doyle et al. 2023). The segregation of PGCs is initiated during oogenesis, wherein germ granules (containing maternal factor *Oskar*) are polarized at the posterior end of the oocyte (Ephrussi and Lehmann 1992; Ewen‐Campen et al. 2012; Rongo et al. 1995). The *Oskar* mutants show disassembled germ granules and fail to form PGCs, resulting in sterile adults (Ephrussi and Lehmann 1992). Furthermore, the accumulation of *Oskar* mRNAs to the anterior pole generates ectopic (at the anterior pole) PGCs that eventually produce functional germline when they are transferred to the normal (posterior) site (Doyle et al. 2023; Ephrussi and Lehmann 1992; Rongo et al. 1995). In *Drosophila*, PGCs bud off continuously from the posterior midgut primordium, involving maternal RNAs, namely, *Germ‐cell less* (*Gcl*), *Nos* (*Nanos*), and *Polar granule component* (*Pgc*), during 1.5–3 h after egg laying (Doyle et al. 2023). Notably, unlike holometabolous *Drosophila*, *Oskar* orthologous gene in cricket (*Gryllus bimaculatus*, an orthopteran insect) has no role in PGC specification (Ewen‐Campen et al. 2012), rather drives neural development (Ewen‐Campen et al. 2013). Furthermore, *Gryllus* lacks maternally inherited germplasm, and PGCs originate in later embryonic stages via induction of abdominal mesoderm (Ewen‐Campen et al. 2013).

*Fishes*—Specification of teleost PGCs requires germline‐specific ribonucleoparticles (RNPs) containing maternally inherited factors like VASA (ATP‐dependent‐DEAD‐box RNA helicase for translation regulation, essential for PGC identity, expansion, migration, and survival), BOULE, DAZ (deleted in azoospermia family), and/or other RBPs, for example, Deleted in Azoospermia Like (DAZL), NANOS, PUM (*Pumilio*), *Piwil‐like* (PIWIL), *Tudor domain–related* (TDRDs), and so forth (Knaut et al. 2000; Köprunner et al. 2001; Raz 2003; Sharma et al. 2024). Segregation of maternally inherited germ granules (total 11 mRNAs [*Vasa, Nanos, Dazl, Dnd1*, etc.] and one noncoding microRNA [miRNA] [*miR‐202‐5p*]) to PGCs is well evident in zebrafish *D. rerio* (Bertho et al. 2021; Raz 2003; Jing Zhang et al. 2017). Germ granules are first identified as large with electron‐dense “nuage” like structures around the onset of somitogenesis, mostly diffused throughout the animal pole/blastodisc, minimal at the yolky vegetal pole (Raz 2003). In fertilized zebrafish eggs, PGCs differentiate during the late blastula stage (at 3 h postfertilization) from four cellular aggregates of germ granules localized to the distal cleavage furrows (Raz 2003). Notably, before zebrafish oogenesis, Buckyball (*Buc*) regulates germplasm assembly by Balbiani formation (Bontems et al. 2009). During the 2–4‐cell stages of zebrafish, *Vasa* and *Nanos* transcripts are detected on the marginal edges of the first two meroblastic cleavage planes (Knaut et al. 2000; Köprunner et al. 2001). By the four‐cell stage, *Vasa* mRNA is embedded within germplasm and appears as an electron‐dense matrix termed nuage (Knaut et al. 2000). In contrast, *Olvas* the *Vasa* homolog of medaka *Oryzias latipes* was found to be uniformly distributed on either dorsal side of the embryonic shield, up to stage 16 of late gastrulation whereas *Nanos* transcripts get diffused (fail to aggregate) at the two first cleavage planes (Herpin et al. 2007; Shinomiya et al. 2000). However, despite such dynamic difference(s) observed in the localization pattern in these mRNAs, the mode of PGC specification remains conserved (Herpin et al. 2007).

*Frogs*—Arunan germplasm is constituted by ribosomes, mitochondria, and electron‐dense particles collectively known as germ granules or “nuage,” with multiple coding and noncoding RNAs (*Xdazl, Xcat_2_, Xvelo1, Vasa*, etc.). Frog germplasm originates from vegetal blastomeres of the presumptive endoderm after holoblastic cleavage at the blastula stage (Houston and King 2000; Porras‐Gómez et al. 2021). PGCs are formed by asymmetric segregation of maternally inherited RNPs into a single‐daughter blastomere up to gastrulation. Notably, transplantation of the vegetal germplasm of *Xenopus* to the animal hemisphere results in the ectopic generation of PGCs, indicating an autonomous capability of germplasm in amphibian germline formation (Tada et al. 2012). The major factor critical for anuran PGC specification is *Xdazl* (codes for *Xenopus* DAZL), which is expressed in germline cytoplasm from blastula up to tail development (Porras‐Gómez et al. 2021; Tada et al. 2012). Notably, *Xdazl* ablation results in PGCs‐deficient tadpoles, indicating its role in both PGC specification and migration (Tada et al. 2012). Other RNPs found in *Xenopus* germplasms are Hermes associated with mRNAs like *Ringo/Spy, Mos, Nanos1* (*Xcat*_*2*_), two splice variants of *Xvelo1* (homologous to *Buc*), and two RBPs, namely, Rbm24b and Rbm42b (Nijjar and Woodland 2013).

*Urodeles*—Urodeles lack maternally inherited typical germplasm; thereby, PGCs do not originate from predetermined cells but rather develop during mesodermal induction from ventral vegetal cells at the blastula stage (Nieuwkoop 1969). In the Axolotl larva of *Ambystoma*, mesodermal cells form PGCs by fibroblast growth factor (FGF), BMP4, and *Brachyury* signaling(s) through the primitive ectoderm of the animal cap (Chatfield et al. 2014). Urodele germ‐cell identity and potential are maintained by *Xdazl* expression and by MAPK signaling, respectively, and germline‐specific irreversible commitment is achieved not before the early stages of tailbud formation (Chatfield et al. 2014; Porras‐Gómez et al. 2021).

*Reptiles*—PGCs generally derived via inductive process exhibiting diverse origins, for example, posterior crescent (the reptilian equivalent of the mammalian primitive streak) in viviparous lizard *Zootoca vivipara*, formerly *Lacerta vivipara*, cells surrounding blastodisc in long‐tailed skink *Mabuya megalura*, slowworm *Anguis fragilis*, chameleon *Chamaeleo bitaeniatus*, tuatara *Sphenodon punctatus* and posterior region of the hypoblast in painted turtle *Chrysemys marginata*, loggerhead sea turtle *Caretta caretta*, common musk turtle *Sternotherus odoratus*, and so forth (Bertocchini and Chuva de Sousa Lopes 2016; Pilato et al. 2013). In the pond slider turtle, *Trachemys scripta*, *Dazl*, and *Vasa* genes have been detected in the presumptive mesoderm cells at the posterior region of the blastopore and in the hypoblast (Bachvarova et al. 2009). In testudines, *Dazl* expression gradually increases from the blastoporal plate of the gastrula to the endodermal layer below the blastopore plate (Bachvarova et al. 2009). Notably, where most reptilian species exhibit an inductive mode of PGC specification, aspic viper snake *Vipera aspis* PGC arises from epiblast cells probably via preformation (Bertocchini and Chuva de Sousa Lopes 2016; Pilato et al. 2013; Porras‐Gómez et al. 2021).

*Birds*—Fowl PGCs derived from the ventral area pellucida of the epiblast area (Kim and Han 2018). The typical localization pattern of chicken *Vasa* homolog (*Cvh*) and *Dazl* in oocytes and embryonic cleavage furrows confirms a preformative mechanism in birds (Lee et al. 2016; Rengaraj et al. 2022; Tsunekawa et al. 2000).

*Rodents*—In mice, EEE and visceral endoderm (VE) induce the posterior‐epiblast (PE) cells to be specified as nascent PGCs on embryonic days (E) 6.25 of prestreak epiblast embryos (Hancock et al. 2021; Mikedis and Downs 2014). In contrast, VE‐derived *Cer1* (*Cerberus 1*) prevents the anterior‐epiblast cells from developing a similar fate (Leitch et al. 2013). Postgastrulation (~ E6.5–7.0), these nascent (PE‐derived) PGCs express *Ifitm3* (Interferon‐induced transmembrane protein 3 or *Fragilis/Mil‐1), Blimp1* (Transcriptional repressor B lymphocyte‐induced maturation protein 1, also known as PR domain‐containing protein1, *Prdm1*), *Hoxb1* (Mikedis and Downs 2014). Subsequently, during neural‐plate to head‐fold stage (~ E7.0–8.0), the bone morphogenetic proteins (*Bmp4* and *Bmp8b* from EEE and *Bmp2* from VE) and transcription factors like *Blimp1* (*Prdm1*), *Prdm14* (PR domain zinc‐finger protein 14), *AP2γ* (activating enhancer‐binding protein 2γ or *Tfap2c*) collectively drives the inductive process for PGC specification by E7.0–7.5 (Kurimoto and Saitou 2019; Leitch et al. 2013; Magnúsdóttir et al. 2013; Mikedis and Downs 2014; Ohinata et al. 2009). ChIP‐seq and single‐cell RNA‐sequencing (sc‐RNA‐seq) analyses suggest that BLIMP1 directly suppresses mitotic and somatic fate genes and further induces AP2γ, which along with PRDM14 triggers the PGC‐specific lineage (Ohinata et al. 2005).

On E7.5, specified murine PGCs do express germline‐specific *Dppa3* (developmental pluripotency associated three, or *Stella*), *Nanos3*, *Ssea1* (stage‐specific embryonic antigen 1), tissue‐nonspecific alkaline phosphatase (*Tnap*), and so forth (Magnúsdóttir et al. 2013). Notably, *Blimp1* (Ohinata et al. 2005) and *Prdm14* (Yamaji et al. 2008) deficient murine PGCs fail to migrate and differentiate appropriately. *Blimp1* suppresses somatic genes (e.g., *Hoxa1*, *Hoxb1*, *Lim1*, *Evx1, Fgf8*, and *Snail*) and stimulates germline‐specific *Stella* (Hancock et al. 2021; Magnúsdóttir et al. 2013). Intriguingly, *Wnt3*‐null (−/−) PE cells fail to express *Blimp1* or *Prdm14* (Lawson et al. 1999; Yamaji et al. 2008), despite being induced by EEE‐derived *Bmp4*. The *Wnt3* signaling augments *Brachyury* (mesodermal transcription factor T) in PE cells, upregulating the expression of *Blimp1* and *Prdm14*, whereas *Bmp4* inhibits *Wnt3‐*driven *Brachyury* (T) to induce mesodermal gene expression in PE (Aramaki et al. 2013). Moreover, *Bmp4‐*induced *Otx2* (Orthodenticle homeobox 2) suppression in PE is critical for murine PGC specification (Jingchao Zhang et al. 2018).

*Pigs*—At fetal age 11–12 days, porcine PGCs originate from PE cells by sequential upregulation of *Sox17* (SRY‐box 17), *Blimp1, Prdm1*, and *Tfap2c* in response to *Wnt* and *Bmp4* signaling from the neighboring extraembryonic mesoderm (Kobayashi et al. 2017).

*Human* **—**PGCs originate from amnion (and PE) during fetal human life/age 11–13 days. The PE cells express EOMES (eomesodermin) followed by TFAP2C, SOX17 (instead of BMP4), and BLIMP1 (but not SOX2 and PRDM14) and get specified to PGCs induced by Activin A (Hancock et al. 2021). Other research groups in recent years have provided evidence with their human gastruloid studies to establish the importance of activin in PGC induction (Etoc et al. 2016; Martyn et al. 2019). Additionally, transcriptional cascades mediated by PAX5–OCT4–PRDM1 are critical in suppressing somatic networks and promoting human PGC programming (Kee et al. 2009; Sybirna et al. 2020). An sc‐RNA‐seq study with 4–19‐weeks‐old foetuses reveals the divergent features of human PGCs, as compared with murine embryos. For instance, the uniqueness of human PGCs includes the reactivation of inactivated X chromosomes by 4 weeks of gestation, maintenance of a uniform transcriptomic landscape between 4 and 11 weeks, and completion of global DNA demethylation by 10–11 weeks (Guo et al. 2015).

## 3 Migration

Embryonic directional movement is synchronized by chemokine signaling that alters the cytoskeletal structure via small GTPases, leading to simultaneous protrusion or adhesion and retraction of leading and lagging edges, respectively (Richardson and Lehmann 2010). Throughout the metazoans, postsegregated/specified PGCs migrate to the site of the developing gonads, where PGCs lose their motility (Richardson and Lehmann 2010). However, as an exception, segregated PGCs of *C. elegans* reach the gonadal site by ingression during gastrulation without any active migration (Pazdernik and Schedl 2013).

*Flies*—In *Drosophila*, PGCs initiate an indirect or passive migration towards the posterior midgut pocket by movements of adjacent tissues from 2–2.5 to 9 h post‐egg‐laying, where E‐cadherin, small GTPase RHO1 and Gβ subunits get localized at the cell periphery in a uniform manner (Jaglarz and Howard 1995). However, by 4.5 h post‐egg‐laying, active migration of PGCs initiates as signaling through *Tre1* (Trapped in endoderm 1, a rhodopsin family G protein–coupled receptor), expressed by these cells leads to polarization followed by redistribution of E‐cadherin, RHO1, and Gβ resulting the dispersal of individual cells from the cluster towards the posterior midgut (Kunwar et al. 2008). Overall, the direction of the migration includes *Tre1‐*dependent transepithelial movement through the midgut followed by Wunen(s) (*Wun*, transmembrane lipid phosphate phosphatases which hydrolyze extracellular phospholipids) and HMGCR (3‐hydroxy‐3‐methylglutaryl coenzyme A reductase) guided bilateral motility of the mesoderm towards the somatic gonadal precursors. The entire event is completed within 4 h (Jaglarz and Howard 1995; Kunwar et al. 2008).

*Fishes*—Distinct patterns of PGC migration have been reported (Blaser et al. 2005; Nishimura and Tanaka 2014; Raz 2003) in teleost fishes. Initially, (i) *Cxcl12* (C‐X‐C motif chemokine 12, previously known as Stromal‐derived factor 1 or *Sdf1a*)—*Cxcr4* (PGC membrane–bound chemokine GPCR CXC motif‐Receptor 4b and *Cxcl12*–*Cxcr7b* (Intracellular CXC motif‐Receptor 7b driven directional migration towards the marginal zone during early gastrulation; subsequently, (ii) passive somatic cell migration by convergent movement, and finally, (iii) *Cxcl12*–*Cxcr4* guided active migration towards posterior end of the lateral‐plate mesoderm (future gonadal site) (Nishimura and Tanaka 2014). Notably, the Deadend 1 protein coded by the *Dnd1* gene governs PGC migration in teleosts (Weidinger et al. 2003). Morpholino‐based inhibition of *Dnd1* results in sterile male phenotype in both zebrafish (Siegfried and Nüsslein‐Volhard 2008; Slanchev et al. 2005) and medaka (Kurokawa et al. 2007) but has no such impact on gonadal phenotype in loach (*Misgurnus anguillicaudatus*) (Fujimoto et al. 2010) and goldfish (*Carassius auratus*) (Goto et al. 2012). Furthermore, inhibiting HMGCR or geranyltransferase (GGT1) disrupts PGC migration in zebrafish (Raz 2003; Richardson and Lehmann 2010).

*Frogs*—*Xenopus* PGCs get specified at the vegetal pole, subsequently undergo passive transport (oriented by extracellular fibronectin) to the animal pole, then follow an active migration (guided by ligand *Sdf‐1* and its receptor *Cxcr4*) into the ventral endoderm and eventually reach the developing genital ridge via the dorsal mesentery (Takeuchi et al. 2010; Whitington and Dixon 1975).

*Reptiles*—The route of migration of PGCs remains inconsistent due to the diverse origin/location of PGCs in reptilian taxa (Bertocchini and Chuva de Sousa Lopes 2016; Pilato et al. 2013).

*Birds*—Chicken PGCs originate at the epiblast, subsequently move ventrally into the hypoblast, and then migrate anteriorly to colonize the germinal crescent. Finally, PGCs reach the forming genital site (GS) via chemokine *Sdf‐1* (in GS and the surrounding mesenchyme)—*Cxcr4* (in PGCs) signaling. Deficiency in *Sdf‐1* and/or *Cxcr4* leads to a severe decline in the gonadal PGCs population (Tsunekawa et al. 2000).

*Rodents*—Murine PGCs are located near primitive streaks on E7.5 and initiate migration posteriorly towards EEE and allantois (Richardson and Lehmann 2010). Thereafter, PGCs migrate across the hindgut to mesoderm directed by c‐Kit–Stem cell factor signaling (Gu et al. 2009), and subsequently, *Cxcl12–Cxcr4* guided bilateral movement facilitates them to reach the gonadal ridge (GR) by E10.5 (Anderson et al. 2000). The colonization of nascent bipotential gonads by PGCs is completed by E11.5 (Ohinata et al. 2009). Notably, *Sox17* inhibits the expansion of the hindgut endoderm and in *Sox17* null (−/−) mice, PGCs are restricted to EEE, failing to reach the GR (Hara et al. 2009). Adhesion molecules like E‐cadherin, β1 Integrin, IFITM1 (interferon‐induced transmembrane protein 1), and so forth, direct the movement of PGCs from hindgut to GR (Kumar and DeFalco 2017; Ohinata et al. 2009). However, it is essential to note here that migratory PGCs continue to express *Nanos3, Oct4* (*Pou5f1*), *Dnd1, Tfap2c*, *Sox2* (*Sox17* for humans), and so forth (Leitch et al. 2010). In posterior mouse gastrula (E6.75–9.0), although *Blimp1* (*Prdm1*) and *Stella* (*Dppa3*) are expressed by nascent migrating PGCs, *Blimp1* has been found to be required for differentiation of presumptive allantois (Mikedis and Downs 2017), whereas *Stella* shown to be involved in mesendodermal differentiation and haematopoiesis at the fetal–placental interface (Wolfe et al. 2017). These data indicate migratory PGCs remain uncommitted to gametogenesis (Mikedis and Downs 2012, 2014; Rey et al. 2000; Mikedis and Downs 2017).

However, PGCs residing at GR gradually start expressing unique transcripts like *Dazl* (deleted in azoospermia‐like, an autosomal homolog of Y‐linked DAZ family), *Ddx4* (DEAD box polypeptide 4, also called mammalian *Vasa* ortholog), *Mael* (murine homolog of a *Drosophila* nuage protein Maelstrom), *Tdrd12* (Tudor domain–containing 12), and so forth (Nicholls et al. 2019).

*Human*—PGCs in human embryos are first detected on fetal days 11–13 and thereafter, start migrating, reaching yolk‐sac on 20–22 days, then in hindgut by 24–26 days and subsequently reach GR for 32–37 days. However, the migration of PGCs (expressing pluripotent markers, viz., *Oct4, Nanog, Klf4, Sall4*, and *Lin28a*) takes around 60 days, and nascent fetal gonads get fully colonized only after 8 weeks of gestation (Rey et al. 2000). The 2.5–6 weeks old PGC markers are as follows: *cKit, Dppa3, Alpl, Nanos3, Sox15, Cdx2, Blimp1, Sox17, Prdm14, Prdm1*, *Tfap2ac*, and so forth, whereas the 7–12 weeks old PGC markers are *Ddx4, Dazl, Tdrd9, Mael, Tex14, Il13ra2*, *Cd38*, and so forth (Julaton and Reijo Pera 2011; Wen and Tang 2019).

## 4 Alteration of PGC Epigenome

Postspecification, the metazoan germline undergoes remarkable epigenetic modifications (by DNA demethylation or methylation of histones) to generate a unique germline‐specific transcriptional programming (Strome and Updike 2015).

*Roundworms* and *flies*—Despite forceful expressions of *chemotaxis 1* (critical for neural fate) and *helix–loop–helix 1* (for muscle) in germ cells of *C. elegans* (either at larval or adult stage), such transgenic germ cells fail to drive somatic fates, indicating a robust germline–specific epigenetic programming (Tursun et al. 2011). MES histone modifiers (e.g., *Mes‐4* for active modification at H3K36me2 and H3K36me3 [di/tri‐methylation at lysine 36 of H3], whereas *Mes‐2, Mes‐3*, and *Mes‐6* [worm polycomb repressive complex 2, PRC2] suppressive at H3K27me3 [tri‐methylation at lysine 27 of H3]) play a critical role(s) in such germline programming. *Mes* deficiency leads to sterility due to massive apoptosis of PGCs (Gaydos et al. 2012, 2014). In adult germline, either specific histone repressors (viz., PRC2, *Spr5* [suppressor of presenilin defect 5], *Let‐418* [lethal 418] [both inhibit H3K4 methylation]) or other proteins (like *Mex‐3* [cytoplasmic RNA‐binding translational regulators like muscle excess 3], *Gld‐1* and *Lin‐41* [abnormal cell lineage 41]) prevents somatic programming (Ciosk et al. 2006). *Gld‐1* and *Gld‐2* potentially regulate the mitotic to meiotic transition/switch, and mutants of *Gld‐1*/*Gld‐2* exhibit somatic fate (Kadyk and Kimble 1998).

Like roundworms, the transcriptional gene silencing of PGCs is mediated by blocking elongation factor P‐TEFb. This cyclin‐dependent kinase phosphorylates the carboxy‐terminal domain (CTD) of RNA Polymerase II in flies (Nakamura and Seydoux 2008). Particularly, germline‐specific *Pie1* (CCCH zinc‐finger protein) attenuates *C. elegans* P‐TEFb, whereas the PGC‐specific polar granule component fulfills the same in *Drosophila* (Ewen‐Campen et al. 2010; Kurimoto et al. 2008). Notably, the RBP NANOS inhibits CTD phosphorylation and somatic gene expression in migratory PGCs of *Drosophila*, sea urchin (*Strongylocentrotus purpuratus*), and *Xenopus* (Ewen‐Campen et al. 2010).

*Fishes* and *mammals*—In zebrafish and mice, *Dnd1* maintains the germline‐specific transcriptomic signature (Ewen‐Campen et al. 2010; Richardson and Lehmann 2010). In mice, during E6.25–8, transcription in PGC gets selectively suppressed (not globally), for example, *Blimp1* inhibits mesodermal development (Kurimoto et al. 2008). However, a global suppression is observed in specified PGCs (E9–10.5) via dephosphorylation of CTD of RNA polymerase II (Saitou et al. 2002; Seki et al. 2007; Shirane et al. 2016; Smith and Meissner 2013). Notably, postspecification, migratory PGCs exhibit two successive phases of demethylation (Kurimoto and Saitou 2019). The first wave remains passive (without involving active DNA demethylases [coded by *Aid*, *Apobec*, or *Tet* genes] and instead operates via suppression of de novo DNA methyltransferases [*Dnmt3a/b*]), occurring between E6.5 and E10.5 (Hargan‐Calvopina et al. 2016; Kagiwada et al. 2012). During this period, DNA methyltransferase 1 (*Dnmt1*) maintains the imprinted loci (paternal or maternal) and the meiotic gene promoters remain functional, whereas *Dnmt1*‐deficient PGCs show precocious/premature germ‐cell development (Larose et al. 2019). A critical heterochromatinizing suppression (e.g., *Setdb1* and *Prmt5* driven H3K27me3 [trimethylation at lysine 27 of H3] or H2A/H4R3me2 [dimethylation at arginine 3 of H2A and H4], respectively) is established in PGCs during E8.5–11.5 (Ng et al. 2013) and blocking of such events triggers germline retroviral activation resulting in sterility (Hackett et al. 2013). The subsequent wave of demethylation coincides with sex determination (during E10.5–12.5), characterized by active DNA demethylation of genes by *Tet1/2* (and other enzymes) (Yamaguchi et al. 2012). This process, therefore, erases the parental imprints and post‐E13.5, around 96% of the germline genome becomes hypomethylated (Kurimoto and Saitou 2019; Larose et al. 2019). Although *Tet1* (5‐position of cytosine [5mC]‐specific dioxygenase) deficiency does not affect the global demethylation in PGCs, it exhibits a significant reduction in oocyte numbers with meiotic defects (Yamaguchi et al. 2012). *Tet1* has been shown to maintain the genome‐wide loss of 5‐methylcytosine (DNA demethylation) in PGCs and plays a notable role during the transition of gametogenic‐competent cells (GCCs) to gonocytes in XY embryos (Hill et al. 2018; Huang et al. 2021). After DNA demethylation, PGCs undergo PRC2‐mediated sex‐specific remodeling of repressive histone modifications (particularly reduction in 5mC and H3K27me3 in males) (Hill et al. 2018). Genetic ablation of *Ezh2* (Enhancer of zeste homolog 2, a functional enzymatic component of PRC2) in mice leads to abnormal transcriptional activation, retrotransposon derepression, and loss of XX germ cells, indicating such chromatin modifications are critical for regulated gene expression, ensuring germ‐cell differentiation (Huang et al. 2021). Notably, in hypomethylated human PGCs (8–9 weeks old), transposable elements L1HS (Line1 retrotransposon), SVA (SINE‐VNTR‐Alu), and olfactory receptor genes maintain a heterochromatin state, where an active network of H3K27me3, H3K9me3, and SETDB1 (Histone‐lysine N‐methyltransferase) critically regulates the temporal control of germline gene expression (Gruhn et al. 2023).

An active contribution of the Nucleosome Remodeling and Deacetylase (NuRD) complex in constructing the germline molecular landscape becomes evident as dedifferentiation of PGCs into pluripotent embryonic germ cells (EGCs) (subsequently to somatic cells) is observed after functional ablation of MBD3 (methyl‐CpG‐binding domain 3, a critical structural component of NuRD complex) (Rais et al. 2013). Notably, this demethylation pattern/event remains conserved in other vertebrates, including humans (Kurimoto and Saitou 2019; Larose et al. 2019).

## 5 Competence of Mammalian PGC by *DAZL*

The developmental competence towards gametogenic (meiotic) commitment acquired by postmigratory, gonad‐resident PGCs (Kurimoto and Saitou 2019), has been extensively examined using genetically manipulated mouse models (Nicholls and Page 2021). The migratory PGCs remain mitotically active, expressing pluripotency markers (e.g., *Oct4*, *Sox2*, and *Nanog*) and colonizing GR/nascent developing gonads by E10.5–11 (Nicholls et al. 2019). Postarrival to the GR/bipotential gonads, PGCs undergo a massive global genomic demethylation during E10.5–12.5 (Kurimoto and Saitou 2019; Larose et al. 2019). This comprehensive epigenetic reprogramming leads to the induction of germ‐cell‐intrinsic RBP, coded by *Dazl* that transforms the gonadal PGCs into GCCs (Lin et al. 2008). This *Dazl‐*induced cellular programming in postmigratory PGCs establishes a unique developmental competence for meiotic commitment (Gill et al. 2011). However, somatic GR also substantially supports such germline competence of PGCs mice, *as Gata‐4* (critical to form the adrenal‐GR complex from the coelomic epithelium) ablation leads to impaired PGC–GCC transition (Hu et al. 2015). Notably, mismigrated PGCs show ectopic (adrenal gland) meiotic entry (at E12.5–13.5) (Upadhyay and Zamboni 1982). The critical role of *Dazl* in determining the development and survival of XY germ cells during E12.5–15.5 becomes evident from knockout (KO) mice studies (Lin and Page 2005). *Dazl* deficiency leads to progressive PGC loss due to apoptosis with age (Chen et al. 2014). Furthermore, a *Dazl* hypomorph model shows a 66% reduction in follicle numbers, highlighting its impact on premature ovarian insufficiency (Rosario et al. 2019). *Dazl* inactivation in human PGCs disrupts miRNA biogenesis, including the let‐7 family (Yan et al. 2022). Intriguingly, the KO of *Dazl* does not restrict PGC arrival to GR/nascent gonads but results in persistent expression of *Nanog, Sox2, Lin28*, and so forth (Gill et al. 2011). Moreover, *Dazl null* (−/−) PGCs develop into germline teratomas (Nicholls et al. 2019). *Dazl* expression continues from the PGC stage throughout the gametogenic progression (Seligman and Page 1998), and *Dazl‐*KO mice (both XY and XX) are infertile (Ruggiu et al. 1997). Furthermore, *Dazl*‐KO testes show impaired A_al_ (aligned undifferentiated spermatogonia A) to A_1_ (transit amplifying differentiating spermatogonia A) transition (Schrans‐Stassen et al. 2001) and defective/abnormal meiotic divisions (Saunders et al. 2003). However, postnatal *Dazl* ablation leads to defects in the differentiation of spermatogonial stem cells, spermatocytes, and spermatids (Saunders et al. 2003). Recently, *Dazl* has been shown to drive a comprehensive translational program in spermatogonial progenitors critical for their expansion and differentiation (Mikedis et al. 2020). Particularly in humans, *Dazl* directs PGC development, whereas *Daz* and *Boule* drive the meiotic progression and functional maturation of gametes (Kee et al. 2009).

Notably, human embryonic stem cell (hESCs) have been shown to lose pluripotency and initiate meiotic division by intrinsic *Dazl* and *Boule*, whereas extrinsic factors (viz., GDF9 and BMP15) induce folliculogenesis (Jung et al. 2017). Like *Dazl*, other germline genes also play critical roles in spermatogenic development (Nicholls and Page 2021). *Mvh* (*Ddx4/Vasa* homolog) null (−/−) mice are infertile with zygotene arrest (Tanaka et al. 2000). Adult *Mael* mutant testes show severe spermatogenic defects, including L1 ribonucleoproteins accumulated in large cytoplasmic enclaves and nuclei of spermatocytes, DNA damage leading to meiotic arrest (Soper et al. 2008). Inactivation of *Tdrd12* (or *Ecat8*) in mice leads to depression of retrotransposons and infertility, indicating the contribution of *Tdrd12* in secondary piRNA biogenesis and transposon silencing in the male germline (Pandey et al. 2013).

## 6 Meiotic Onset

In mice, sex determination initiates around E11.5 by triggering the *Sry* gene in SF‐1/Nr5a1^+^ XY somatic cells to induce male programmed genetic (*Sox9*, *Amh*, *Fgf9*, *Dmrt1*, etc.) cascades which drive the testicular morphogenesis (DeFalco et al. 2014; Koopman et al. 2016). However, during E12.5–13.5, the XX‐GCCs enter into meiotic prophase by upregulating *Stra8* (stimulated by retinoic acid [RA] gene 8) and *Rec8* (Meiotic recombination 8) under the influence of RA in the fetal ovary (which gets differentiated by *Wnt4/Rspo1*) (Bhattacharya et al. 2022; Bowles et al. 2016, 2006; Endo et al. 2019; Griswold et al. 2012; Koubova et al. 2014, 2006; Spiller et al. 2017; Tedesco et al. 2013). In contrast, during E12.5–13.5, RA gets degraded by CYP26B1 in fetal testes (Bowles et al. 2006; Koubova et al. 2006; MacLean et al. 2007), preventing meiotic entry of XY germ cells, which get arrested at the *G*_0_/*G*_1_ stage termed as gonocytes/prospermatogonia (Bhattacharya et al. 2022; Endo et al. 2019; Griswold et al. 2012). Intriguingly, CYP26B1 was found to be critical for fetal testicular development (persistent presence of RA signaling in XY *Cyp28b1* null [−/−] embryos augments *Dax1* expression that blocks steroidogenesis and *Amh* expression leading to impaired male genital track development) (Bowles et al. 2018) and spermiation during adulthood (Hogarth et al. 2015). Notably, on E13.5, *Cyp26b1* expression gets downregulated; however, XY germ‐cell‐intrinsic *Nanos2* prevents their meiotic entry (Suzuki and Saga 2008). Ectopic meiosis (upregulating *Stra8*) has been found in *Nanos2*‐null XY germ cells on E14.5 (Suzuki and Saga 2008).

**Figure 2:**
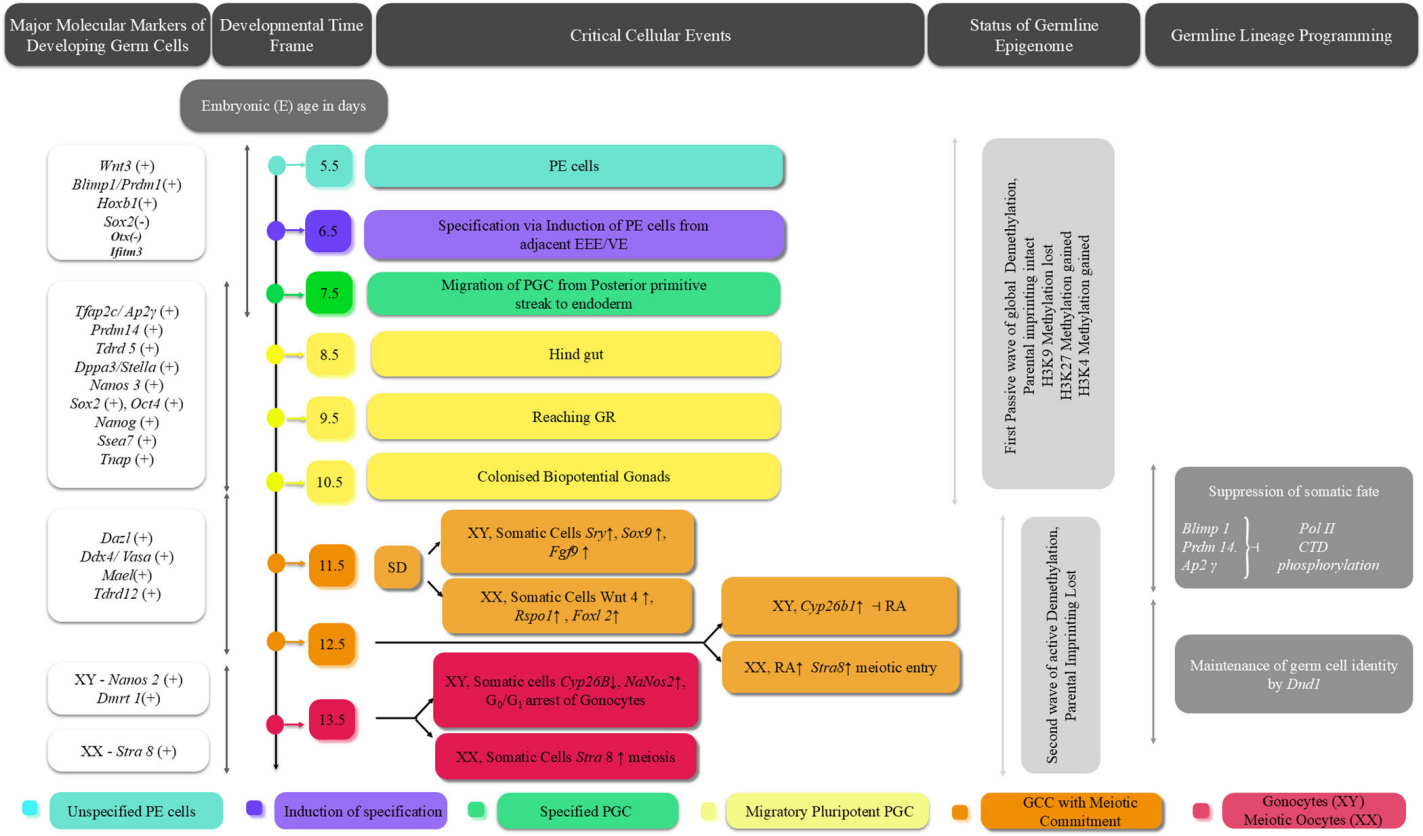
The developmental events occurring in murine primordial germ cells (PGCs) from origin to specification, and migration to commitment. On E7.5, murine posterior‐epiblast cells (PEs) get specified into nascent PGCs by induction from adjacent extraembryonic ectoderm (EEE) and visceral endoderm (VE). The critical players in this event are the EEE and VE‐derived *Bmp4, Wnt3, Blimp1, Prdm1*, and *Tfap2c*. During embryonic days (E) 7.5–10.5, specified PGCs (which are mitotically active and express pluripotent markers) migrate through the hindgut and colonize at the gonadal ridge (GR)/bipotential gonads. During E10.5–12.5, all gonadal PGCs lose their pluripotency and transform into gametogenic competent cells (GCCs) to gain meiotic commitment by *Dazl*. On E11.5, sex determination gets triggered by the *Sry* gene from somatic cells of XY gonads, leading to testicular development (upregulating *Sox9, Fgf9*, and *Dmrt1*), whereas XX somatic cells upregulate *Wnt4, Rspo1*, and *Foxl*_*2*_) for ovarian development. On E11.5–12.5, retinoic acid (RA) gets degraded by *Cyp26b1* only in XY gonads, therefore restricting the meiotic entry of XY‐GCCs, whereas XX‐GCCs enter into meiosis via upregulating *Stra8* induced by RA. The first wave of passive demethylation happens during E6.5–10.5, while the second wave of active demethylation occurs during E11.5–13.5. Suppression of somatic fate in the germline is mediated by phosphorylation at the carboxy‐terminal domain (CTD) of polymerase II by *Blip1, Prdm14*, and *Tfap2c. Dnd1* maintains the germline‐specific identity. On E13.5, *Cyp26b1* expression declines in XY gonads; however, upregulation of *Nanos2* prevents the meiotic entry of XY germ cells and ensures mitotic arrest at the *G*_0_/*G*_1_ state, forming gonocytes. FGF, fibroblast growth factor.

**Figure 3:**
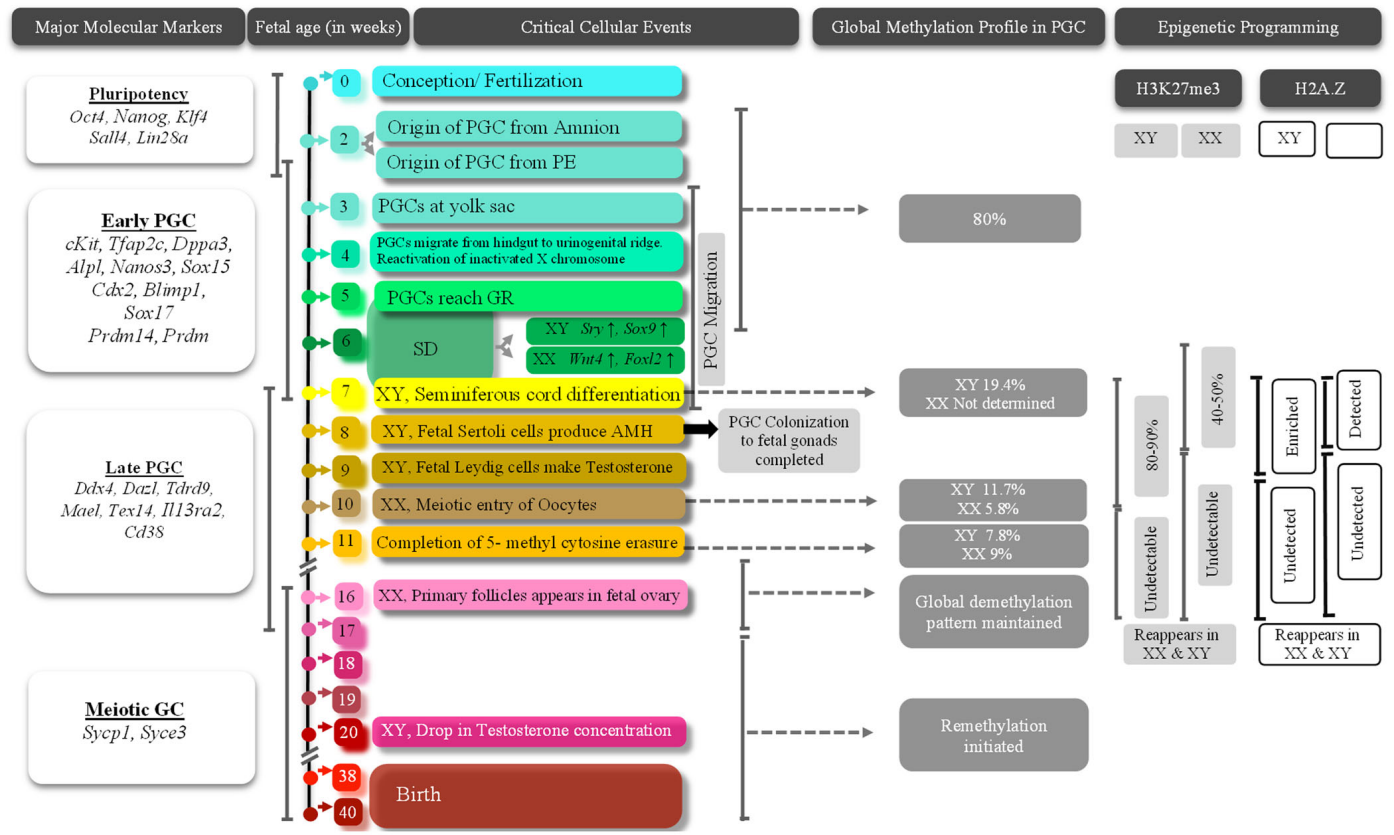
Comparable developmental events observed in human PGCs. Notable species differences are evident in terms of duration of time scale (in weeks of gestation), variable PGC markers, and occurrence or degree of global demethylation profiles. GC, germ cell; GR, gonadal ridge; PE, posterior‐epiblast; PGC, primordial germ cell.

Table 1 summarizes the cellular and molecular events of specification, migration, and developmental commitment of metazoan PGCs, whereas Figures 2 and 3 illustrate the ontogeny of murine and human PGCs, respectively.

**Table 1:** Cellular and molecular details of specification, migration, and developmental commitment of metazoan primordial germ cells (UI = unidentified).

S. No.	Taxa	Site of origin	Developmental stage	Specification mode	Maternal intrinsic factors	External inducing factors	Molecules guiding migration	Pluripotent markers	Germ‐cell–specific markers	Factors for germline commitment
1	*Nematode worm*									
	*Caenorhabditis elegans* 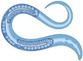	First cleavage 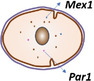	First cleavage blastomere 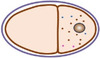	Preformation	*Etr1, Gcl, Mag1, Mes‐2, Mes‐3, Mes‐4, Mes‐6, Mex‐1, Mex‐3, Nanos, Par‐1, Pie‐1, Vasa, Pufs, Pgl1, Pgl3, Glh‐1, Glh‐4*	UI	UI	UI	*Prg1, Prg2, Csr1, Lin‐41*	*Gld‐1, Gld‐2*
2	*Arthropod insect*									
	*Drosophila melanogaster* 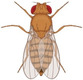	Early cleavage/late embryogenesis 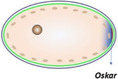	Early cleavage blastomere/mesoderm 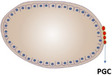	Preformation	*Oskar, Boule, Aubergine, Gurken, Gustavus, Vasa, Tudor* *Homeless, Mago nashi, Bruno, Valois, Capuccino, Germ‐cell‐less, Nanos, Orb, Par‐1, Pgc1, Pumilio, Spire, Staufen, Tropomysin II, Torso*	UI	*Tre1 (Cg3171), E‐cadherin, Fpps, Wunen, Wenen2, Hmgcr, Ggt1, Pum*	*Nos*, Thickveins	*Vasa*	UI
3	*Teleost fish*									
	*Danio rerio* 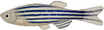	Distal cleavage furrows 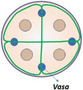	Late blastula stage (at 3 h postfertilization) 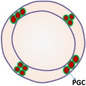	Preformation	*Nanos, Vasa, Dnd1, Bucky ball, Dazl, Z‐otu*, *miR‐202‐5p*	UI	*Rho1, Sdf‐1* (*Cxc12*), *Cxcr4, Cxcr7b, E‐cadherin, G proteins*,	*Nanog, Oct4/Pou5f3, Sox B*	*Vasa, Nanos3, Cbx2, Amh, Dmrt1, Ly75/CD205*, carbonic anhydrase (*Ca15b*)	UI
4	*Amphibia*									
	*Frog Xenopus laevis* 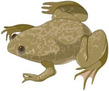 *Urodele* *Ambystoma* 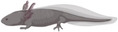	Presumptive endoderm of vegetal blastomere 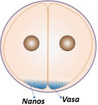 Ventral vegetal cells 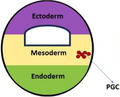	Blastula 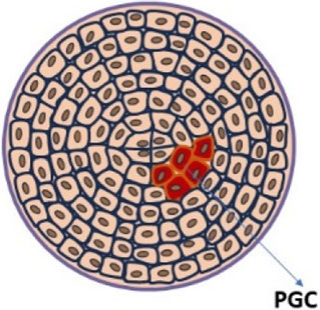 Blastula, (germline‐specific commitment during tailbud formation) 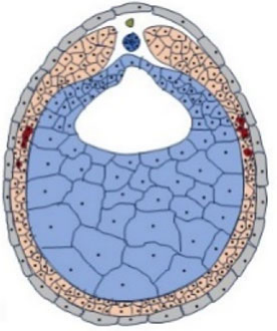	Preformation Epigenesis	*Xdazl, Xcat*_*2*_, *Xvelo1, Vasa* *Vg1, VegT, Xwnt11*. UI	UI *Fgf4, Bmp4, Brachyury*	*Sdf‐1, Cxcr4, Irx5* UI	*Pouv* genes *Xlpou25* (*Pou5f3.2*), *Xlpou60* (*Pou5f3.3*), *Xlpou91* (*Pou5f3.1*), *Nanog*. *PouV, Oct4/Pou5f3.1*	*Xdazl* *Xpat, Germes*. *Vasa, Dazl*	UI UI
5	*Reptiles*									
	Testudines *Trachemys scripta* 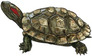	Posterior region of the hypoblast 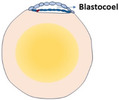	Gastrulation 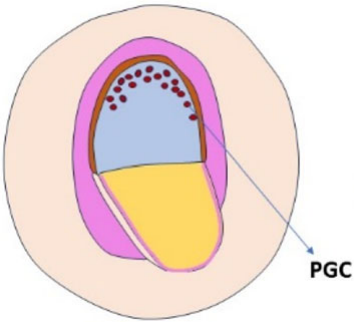	Epigenesis	UI	UI	UI	UI	*Dazl, Vasa*	UI
6	*Birds*									
	*Gallus domesticus* 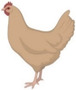	Ventral area pellucida of the epiblast area 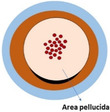	Eyal‐Giladi and Kochav stage‐III 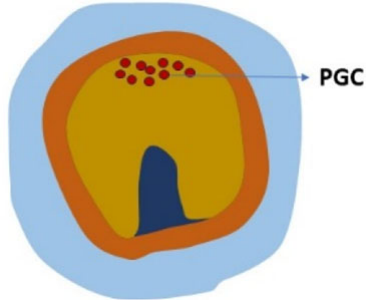	Preformation	*Cvh (Vasa homolog), Dazl*	UI	*Sdf‐1/Cxcl12Cxcr4*	cNanog cPouV	*Dazl, DDX4/Vasa, Dmrt1*	UI
7	*Mammals*									
	*(I) Mus musculus* 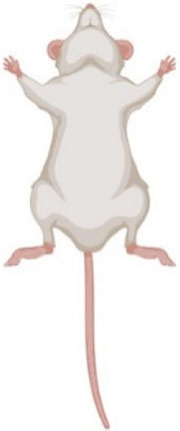 *(II) Homo sapiens* 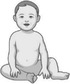	Posterior epiblast 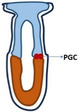 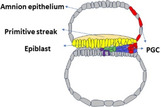 Amnion, posterior epiblast	Onset of primitive streak formation E6.5 days 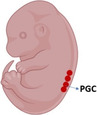 Fetal age 11–13 days Before primitive Streak formation 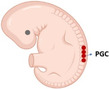	Epigenesis Epigenesis	*Boule, Fragilis, Germ‐cell‐less, Vasa* *Gp130, Mago nashi, Nanos, Par‐1, Pog, Pumilio, Stella.* *Dazl, Boule, Nanos3*	Extraembryonic ectoderm (EEE)–derived *Bmp4, Bmp8* and visceral endoderm–derived *Bmp2, Cer1*, and posterior‐epiblast–derived *Wnt3, Blimp1, Prmd14, Otx2*. EEE‐derived *Activin*, Amnion‐derived *Pax5, Oct4*, posterior‐epiblast–derived *Eomes, Blimp1, Ap2γ, Sox 17*	*Sdf‐1 (Cxcl12), Cxcr4, c‐Kit (Kit), Steel (Kitlg), E‐cadherin, Integrin β1* *Sdf‐1 (Cxcl12), Cxcr4, c‐Kit (Kit), Steel (Kitlg), E‐cadherin, Integrin β1*	*Nanog, Pou5f1/Oct4, Sox2* *Nanog, Pou5f1, Trim‐nhl, Ssea4*	*Dazl, Stella/Dppa3* *Ssea7, Tnap, Ddx4/Vasa*. *Dazl, Ddx4/Vasa*	Cell‐autonomous *Dazl* GR‐dependent *Gata 4* UI

### 6.1 Developing PGCs In Vitro

#### 6.1.1 Rodent Model

In 2009, Hayashi and Surani demonstrated the efficacy of murine epiblast stem cells (EpiSCs) in studying germ‐cell lineage and epigenetic reprogramming. EpiSCs (isolated from E6.5 days old VE) were cultured (in N2B27 medium with 20% knockout serum replacement (KSR), 2 ng/mL recombinant human activin A, and 12 ng/mL basic fibroblast growth factor [bFGF]) to maintain their pluripotency and self‐renewal capacity and further get differentiated into PGCs (with 500 ng/mL BMP4, 250 ng/mL Noggin, and 1.25 μg/mL Chordin cocktail) by 48 h. Intriguingly, these Stella^+^ EpiSCs‐derived PGCs get induced to undergo dedifferentiation in 5–6 days of culture (in Dulbecco's Modified Eagle Medium having 15% Fetal Calf Serum (FCS), nonessential amino acids, 2 mM l‐glutamine, penicillin/streptomycin, 1 mM sodium pyruvate, 50 μM 2‐mercaptoethanol with 1000 U/mL LIF, and 25 ng/mL bFGF supplementations) into pluripotent EGCs that share comparable epigenetic signature with inner cell mass or embryonic stem cells but not that of the EpiSCs (Hayashi and Surani 2009). In subsequent studies, pregastrulating epiblast‐like cells (EpiLCs) were developed in vitro from ES and/or induced pluripotent stem (iPS) cells using Activin A, bFGF, and 15% KSR. Hayashi et al. (2011) have reconstituted these EpiLCs further to *Ssea‐1* and *Integrin‐β3* expressing PGCLCs by a morphogen cocktail (BMP4, BMP8b, SCF, LIF, and EGF). Finally, these PGCLCs successfully exhibit complete spermatogenesis (Hayashi et al. 2011) and oogenesis (Hayashi et al. 2012) once being transplanted into respective murine gonads. The same group further demonstrated the generation of EpiLCs via simultaneous overexpressions of three transcription factors, for example, *Blimp1* (*Prdm1*), *Prdm14*, and *Tfap2c* (*AP2γ*) in ES (BVSCR26rtTA) cells for 36 h (Nakaki et al. 2013). These EpiLCs subsequently differentiate (when induced by BMP4, BMP8a, SCF, LIF, and EGF) into PGCLCs and finally form spermatozoa (Nakaki et al. 2013). Notably, *Prdm14* alone (without *Blimp1* or *Tfap2c*) is most critical for generating the PGC‐like state in EpiLCs by augmenting *Blimp1* and *Tfap2c* expression, inducing core PGC genes and preventing neural induction (Nakaki et al. 2013). A similar protocol has also been deployed to generate rat ES cells–derived PGCLCs capable of producing functional spermatozoa (Oikawa et al. 2022).

#### 6.1.2 Human Model

The peripheral mononuclear blood cells–derived human iPS cell lines (585A1 and 585B1 expressing *Oct4/Pou5f1, Nanog*, and *Sox2*) have been supplemented by Activin A and Chiron (glycogen synthase kinase [GSK] 3 inhibitor, Wnt agonist) to generate incipient mesoderm‐like cells (iMeLCs) showing expression of *Sp5, Nodal, Mixl1*, and *Eomes* (Sasaki et al. 2015). These iMeLCs further differentiate (with BMP4, SCF, LIF, and EGF treatments) into PGCLCs showing robust expressions of *Sox15, Sox17, Tfap2c, T, Blimp1*, *Prdm14*, epithelial cellular adhesion molecule (*Epcam*), and *Integrin*‐α6, like that of premigratory PGCs found in vivo (Sasaki et al. 2015). Irie et al. (2015) have used *Nanos3*‐mCherry reporter gene expressing human ES cells to demonstrate in vitro differentiation (by BMP2/BMP4, LIF, SCF, EGF, and Rho‐kinase [ROCK] inhibitor [Y‐27632] in the presence of bFGF, TGFβ, and 1% KSR medium) of PGCLCs. These PGCLCs too are found to be transcriptionally comparable with the PGCs (CD38 being a common stable surface marker) collected from human foetuses. Furthermore, unlike mice, *Sox17* (essential for endoderm lineages) acts upstream of *Blimp1* and is found to be most critical in regulating human PGCLC fate (Irie et al. 2015). Similarities between the in vitro generation of murine and human PGCLCs have been reported by Sugawa et al. (2015), where human ES (H9 and HuES6) and iPS cell lines (393.2 and SA8/25) have been reported to differentiate into heterogeneous mesoderm‐like cell (MLC) population and subsequently (when supplemented with Activin A, BMP4, bFGF, and Y‐27632) forming the PGCLCs with minimal *Prdm14* expression. Kojima et al. (2017) have generated PGCLCs in vitro from human iPS cells (585B1 BTAG [46, XY])–derived MLCs via *Eomes*‐mediated *Sox17* induction, followed by *Tfap2c* and *Blimp1* expressions. Intriguingly, rhesus monkey‐based iPS cells (riPSC89 and riPSC90)–derived naïve PGCLCs exhibit differentiation up to *Ddx4* (*Vasa*)‐positive state (but not *Eno2*‐positive spermatogonia) after being transplanted into irradiated testes of either nude mice or rhesus macaques (Sosa et al. 2018). Using xenogeneic reconstituted ovaries, the maturation of female hPGCLCs into premeiotic oogonia‐like cells has also been successfully demonstrated (Yamashiro et al. 2018). Hwang et al. (2020) have reconstituted in vitro differentiation of PGCLCs into mitotically sluggish T1 prospermatogonia‐like cells (T1LCs), showing comparable transcriptomic signatures that have been found in human gonocytes/T1 prospermatogonia in vivo. Furthermore, adopting a CRISPR‐based loss‐of‐function screening test, *TCL1A* (T‐cell leukemia/lymphoma protein 1A) has been identified as a critical regulator of protein synthesis and cell proliferation through protein kinase B‐mammalian target of rapamycin (AKT‐mTOR) signaling in human PGCLCs (Hwang et al. 2023). A recent study showing in vitro differentiation of human iPS cells to PGCLCs indicates long noncoding RNA *Lnc1845* regulates the expression of *LHX8* (a critical inducer for ovarian follicle development) via modulating histone (at H3K4me3 and H3K27Ac) modifications (N. Wang et al. 2023). All these data collectively generate a potential scope for in vitro gametogenesis for fertility restoration/preservation in mammals (Canovas et al. 2017; Hikabe et al. 2016; Makar and Sasaki 2020; Saitou and Hayashi 2021; Yamashiro et al. 2018; Yin et al. 2020; Yu et al. 2023).

Table 2 summarizes the recent success in generating PGCLCs in vitro for (A) murine and human (B) models.

**Table 2:** Recent progress in vitro generation of PGCLCs in (A) rodent models and (B) primate models.

Year	Species	Precursor cell	Treatment cocktail and duration	Transition state	Culture condition and duration	Markers studied for PGCLCs	Development functional into gametes	References
2011	Mouse	ESC and/or iPSC	Activin A, Basic Fibroblast 314 Growth Factor (bFGF) and 15% knockout serum replacement (KSR), PD0325901, CHIR99021, LIF	ESCs and iPSCs to PGCLCs	Morphogen cocktail (BMP4, BMP8B, SCF, LIF, EGF)	Ssea‐1/Integrin β3	Yes—After being transplanted to murine gonads	Hayashi et al. (2011, 2012)
2013	Mouse	iPSCs	Doxycycline (Dox) and Cytokines/Duration: (2–6 days)	EiPLC to PGCs	BMP4, BMP8A, SCF, LIF, EGF	BLIMP1, TFAP2C, PRDM14	Yes—PGCLCs were injected into seminiferous tubules/were used for intracytoplasmic sperm injection into wild‐type oocyte	Nakaki et al. (2013)
2016	Mouse	PSC	αMEM with BMP15, EGF, GDF9, ICI182780	iPSC to PGCs	αMEM supplemented with FSH, BMP15, EGF, GDF9	BV/SC reporter constructs	Yes—The study involved transplanting the in vitro‐generated oocytes into mice to produce offspring	Hikabe et al. (2016
2020	Mouse	mESC	2i + LIF medium	PGCLCs to SSCs	2i + LIF medium	PRDM1, STELLA, NANOS3, PRDM1, PRDM14, NANOS3	—	Yin et al. (2020)
2022	Rat	rESC/rPSC	N2B27 medium with 5% KSR	rPSC to rPGCLC	PGCLC cocktail (BMP4, SCF, LIF, EGF)	Tfap2c, Oct3/4, Sox2, DDX4	Yes—After being transplanted into seminiferous tubules	Oikawa et al. (2022)
2023	Mouse	PSC	FGF2, Activin A, CHI99021 (48 h),	Intermediate pluripotent state	GK15 medium with BMP4, SCF, EGF	Ssea‐1/Integrin β3	—	Yu et al. (2023)

Year	Species	Precursor cell	Treatment cocktail and duration	Transition state	Culture condition and duration	Markers studied for PGCLCs	Development into functional gametes	References
2015	Human	hiPSCs‐derived incipient mesoderm‐like cell (iMeLC)	Activin A with 10% KSR/CHIR00921	hiPSCs to iMeLCs to hPGCLCs	StemFit medium (BMP4, SCF, EGF, LIF) for the first stage 48 h for iMeLC state. Then 2–4 days for the final hPGCLC induction	EpCAM‐BTAG, BLIMP1, TFAP2C, PRDM1, SOX17, SOX15, KLF4, KIT, TCL1A, DND1	—	Sasaki et al. (2015)
2015	Human	hESCs	N2B27 medium supplemented with 1% KSR, bFGF, TGF‐β1, Activin A, Rho‐kinase (ROCK) inhibitor, (48 h)	hiPSCs to hPGCLCs	BMP4/BMP2, LIF, SCF, EGF, ROCK inhibitor	CD38, NAN0S3, BLIMP1, SOX17, SOX2, OCT4	—	Irie et al. (2015)
2015	Human	hESC and hiPSC	N2B27 Medium with Activin A, BMP4, bFGF, Rock Inhibitor (48 h)	hiPSCs to hPGCLCs	GK20 medium containing BMP4, LIF, and Y‐27632	BLIMP1, STELLA, OCT4, NANOG, SOX2, T (mesodermal factor)	Yes—After being transplanted into murine gonads	Sugawa et al. (2015)
2017	Human	iPSCs	Activin A, CHIR99021, Y‐27632 (48 h), GK medium with 15% KSR, 0.1 mM NEAA, 2 mM l‐glutamine, 1 mM, sodium pyruvate, and 0.1 mM 2‐mercaptoethanol	hiPSCs to hPGCLCs through an iMeLC intermediate state	BMP4, SCF, EGF, LIF (up to sixth day of induction)	EpCAM, INTEGRINa6, BTAG	—	Kojima et al. (2017)
2018	Monkey	iPSC	15% KSR, 0.1 mM nonessential amino acids (Life Technologies), penicillin/streptomycin/l‐glutamine, Primocin (Invitrogen), 0.1 mM β‐mercaptoethanol, sodium pyruvate, Activin A, CHIR99021, Y‐27632 (24 h)	iPSC to PGCLC	PGCLC media with BMP4, EGF, LIF (cultured for 15 days)	PRDM1, SOX17, TFAP2C, VASA, OCT4, SOX2, BMP4	Yes—After being transplanted into murine gonads	Sosa et al. (2018)
2018	Human	hiPSCs	N2B27 medium with CHIR99021 and LIF	hiPSCs into hPGCLCs	BMP4, EGF, LIF	BLIMP1, TFAP2C, VASA, SOX 17	—	Yamashiro et al. (2018)
2020	Human	hiPSCs	StemFit Basic Medium with FGF, ROCK inhibitor (24 h)	hiPSCs into hPGCLC, further differentiation of those into M‐prospermatogonia‐like cells (MLCs) and T1 prospermatogonia‐like cells (T1LCs)	StemFit Basic 04 Medium with Y‐27632	NANOG, SOX17, TFAP2C, DAZL, DDX4, POU5F1	—	Hwang et al. (2020)
2023	Human	hiPSCs	GK medium with 15% KSR, NEAA, l‐glutamine, sodium pyruvate, mercapto ethanol, ACTA, CHIR99021	hiPSCs into hPGCLCs	GK15 medium containing BMP4, LIF, EGF, and Y‐27632 (5 days)	SOX17, ITGA6, PDPN, POU5F1, NANOG, SOX2, BLIMP1, SOX 17, TFAP2C, NANOS3	—	N. Wang et al. (2023)
2023	Human	hESCs	hESC medium KODMEM with 20% KSR, 1 mM l‐glutamine, 0.1 mM nonessential amino acids, and 8 ng/mL recombinant human basic FGF	hiPSCs to hPGCLCs	Differentiation medium (KODMEM supplemented with 10% FBS, 1 mM l‐glutamine, 0.1 mM nonessential amino acids, 50 ng/mL BMP4 and BMP8a. Differentiated hESCs were incubated with a differentiation medium containing 50 ng/mL BMP4 and BMP8a for 12 days	LNC1845, LHX8	—	N. Wang et al. (2023)
2023	Human	PSC	FGF2, Activin A, 15% KSR, NEAA, sodium pyruvate, 2‐mercapto ethanol, l‐glutamine	Intermediate pluripotent state	GK15 medium with BMP4, SCF, EGF, ROCK inhibitor	IntegrinA6, epCAM	—	Yu et al. (2023)

Abbreviations: BMP, bone morphogenetic protein; ESCs, embryonic stem cells; FGF, fibroblast growth factor; iPS, induced pluripotent stem; iPSC, induced pluripotent stem cell; hiPSCs, human induced pluripotent stem cells; PGC, primordial germ cell; PGCLCs, PGC‐like cells; PSC, pluripotent stem cell; LIF, leukemia inhibitory factor; mESCs, Mouse embryonic stem cells; rPSCs, retinal‐pigment epithelial stem cells; rESCs, resident primary skin cells; GK, Glasgow's Minimum Essential Medium supplemented with 15% KnockOut™ serum replacement; NEAA, non‐essential amino acids; KODMEM, knockout DMEM; EiPLC, epiblast‐induced PLC; SSC, spermatogonial stem cell; FSH, follicle stimulating hormone; SCF, stem cell factor; GDF, growth differentiation factor; SOX, Sry‐related HMG box; PRDM, PRDI‐BF1 and RIZ1 homology domain containing.

## 7 Concluding Remarks

Evolution of the metazoan germline has been found to be sporadic and stochastic with diverse modes of PGC specification (preformation and epigenesis) adopted by different taxa having apparent phylogenetic convergence (Bertocchini and Chuva de Sousa Lopes 2016; Extavour and Akam 2003; Hansen and Pelegri 2021; Kumar and DeFalco 2017; Strome and Updike 2015). For example, (I) individual species (e.g., orthopteran cricket *Gryllus*, lobe‐finned Sarcopterygiian coelacanth Latimeria and lungfishes, urodele *Ambystoma*, etc.) exhibiting inductive epigenesis within a single taxon which generally follows an inheritable preformation strategy (e.g., insects, fishes, and amphibians) (Johnson et al. 2003; Johnson et al. 2011). (II) Maternally inherited and differentially distributed germ granules get aggregated at the furrows of developing embryos in fishes, amphibians, and birds (D'Avino et al. 2005). Furthermore, most germplasm RNAs/RNPs show a typical perinuclear localization forming the nuage, which subsequently influences the zygotic gene expression and ensures a unique transcriptional identity for the germline (Kulkarni and Extavour 2017). (III) Multiple maternally inherited genes (e.g., *Dazl, Vasa, Dnd1, Nanos*, etc.) are found to be evolutionarily conserved, having a common/shared role in PGC specification, migration, and survival with minor exceptions (e.g.,*Oskar* in flies and cricket) (Extavour and Akam 2003; Strome and Updike 2015). (IV) BMP(s) signaling(s) found to be conserved for inductive PGC specification among different taxa, for example, tunicates, urodeles, testudines, mammals, and so forth (Hansen and Pelegri 2021; Johnson et al. 2011). (V) Acquiring somatic fate of ectopic PGCs (due to mismigration and/or experimental transplantation) indicates that postspecified/segregated PGCs remain developmentally uncommitted towards gametogenesis throughout the metazoan lineage (Extavour and Akam 2003; Nicholls and Page 2021; Richardson and Lehmann 2010; Strome and Updike 2015). (VI) *Dazl* appears to be one of the most critical factors for meiotic commitment (germline licensing) in multiple species from fishes to mammals (Bertho et al. 2021; Hansen and Pelegri 2021; M. Li et al. 2016; Strome and Updike 2015; Tada and Orii 2015). (VII) The molecular landscape necessary for the epigenetic programming (e.g., P‑TEFb‐mediated transcriptomic silencing of metazoan PGCs) unique to germ cells remains phylogenetically comparable (Kurimoto and Saitou 2019). (VIII) An evident chromatin remodeling pattern (global reduction of genomic methylation, elevated H2K27me3, declined H3K9me2, etc.) has been found to be consistent among diverse animal taxa with minor deviations (Lesch et al. 2016).

## 8 Future Directions

The generation of mammalian PGCLCs in culture has been a remarkable achievement in the field of reproductive biology, assuring a potential promise for in vitro differentiation of functional gametes. Furthermore, studying PGC development will decipher the molecular mechanisms underlying the formation of germ‐cell tumors (Nicholls et al. 2019). For example, misdirected PGCs or PGCs having mutated *c‐Kit* and/or *Kras* spontaneously develop/generate either ectopic tumorigenesis or carcinoma in situ, respectively (Upadhyay and Zamboni 1982). In summary, studying metazoan PGCs has extensively enriched our fundamental concepts of cellular development with respect to phylogenetic relevance and clinical significance. Despite all, most of the available information/data on metazoan germline, however, are limited to model organisms (like nematode worm *C. elegans*, fruit fly *D. melanogaster*, teleost fish *D. rerio*, anuran amphibian *X. laevis*, and eutherian mammal mice *M. musculus*) only. Therefore, to comprehend the randomness of the metazoan phylogeny, future investigation should focus on nonmodel species deploying multiomics‐based cutting‐edge technologies like next‐generation sc‐RNA‐seq and CRISPR/CAS9 genome editing (T. Li et al. 2023).

## Author Contributions

**Indrashis Bhattacharya:** conceptualization, funding acquisition, writing – original draft, supervision, data curation. **Lakshmi K. Nalinan:** formal analysis. **K. V. Anusree:** formal analysis. **Ahmed Saleel:** formal analysis. **Aditi Khamamkar:** formal analysis. **Souvik Dey:** conceptualization, funding acquisition.

## Data Availability

The data that support the findings of this study are openly available in PubMed at https://pubmed.ncbi.nlm.nih.gov/.
